# A 10-year Retrospective Review of Botulinum Toxin Injections and Surgical Management of Sialorrhea

**DOI:** 10.7759/cureus.7916

**Published:** 2020-05-01

**Authors:** Rachel E Weitzman, Kosuke Kawai, Roger Nuss, Amy Hughes

**Affiliations:** 1 Otolaryngology, Harvard Medical School, Boston, USA; 2 Otolaryngology, Boston Children’s Hospital, Boston, USA; 3 Otolaryngology, Boston Children's Hospital, Boston, USA; 4 Otolaryngology, Connecticut Children's Medical Center, Hartford, USA

**Keywords:** sialorrhea, drooling, salivary gland surgery, botox injections, pediatric otolaryngology

## Abstract

Background

Sialorrhea is a common comorbidity among children with neurologic disorders. Botulinum toxin injections and surgical procedures are recommended for the management of pathological sialorrhea in patients who fail conservative management or with concerns for salivary aspiration. The following review evaluates outcomes following botulinum toxin injections and surgical interventions for sialorrhea over a 10-year period with a focus on treatment options and outcomes for patients with anterior and posterior drooling.

Methods

The study included all patients less than 25 years of age who underwent a procedure for drooling (Current Procedural Terminology (CPT) codes 42440, 42450, 42509, 42510, 64611 matched with the International Classification of Diseases (ICD)-9 and ICD-10 codes 527.7 and K11.7) from January 1, 2006 to December 31, 2015. A chart review collected demographics, drooling medication use, and type of drooling (anterior, posterior, both). Outcome variables included pre- and post-procedure number of bibs, parent-reported outcomes, post-intervention drooling medication requirement, post-procedure length of stay, and complications.

Results

Seventy-one patients were included in our analysis, with 88 total procedures performed. The average age at first intervention was 8.9 years; 43 patients were male and 40 patients had cerebral palsy. Thirty-one patients experienced posterior drooling or anterior/posterior drooling. These patients were more likely to undergo surgery as the first invasive intervention. The most commonly performed interventions were botulinum toxin injections (28 patients, 39%) and sublingual gland excision (SLGE) with submandibular duct ligation (SMDL) (36 patients, 51%). Improvement following injections was noted in 56% of patients versus 73% of patients following any surgical intervention.

Conclusion

Management of drooling is complex with 18 different procedures performed over 10 years. Surgical interventions, specifically SLGE with SMDL and submandibular gland excision (SMGE), result in substantial improvement; these are commonly performed as the first intervention in patients with posterior drooling. By reviewing our experience, we hope to guide management decisions and help manage patient and caregiver expectations.

## Introduction

Sialorrhea is a common concern among children with neurological disorders. The estimated prevalence rate among individuals with cerebral palsy is 40% [1]. Sialorrhea results from failed clearance and removal of saliva from the oral cavity, most often associated with impaired swallowing. Less commonly, patients may have increased production of saliva (usually idiopathic or drug-related). The parotid, submandibular, and sublingual glands are defined as the major salivary glands and are targets for intervention.

Sialorrhea may be further categorized as anterior or posterior. Anterior sialorrhea is defined as saliva loss anteriorly from the mouth with visible spilling into the lip area and chin, while posterior sialorrhea occurs when saliva spills and pools from the oropharynx into the hypopharynx. The former typically leads to psychosocial and physical complications, including impaired self-esteem, damage to clothing and educational materials, increased work for care-givers, poor dentition, and perioral infections. The latter is associated with more significant medical complications related to salivary aspiration including recurrent respiratory infections and chronic lung disease. 

Patients with sialorrhea benefit from coordinated care between multiple specialties, which may include neurology, developmental pediatricians, chronic care specialists, otolaryngology, dental, and physical medicine and rehabilitation. This allows for comprehensive assessment and treatment of drooling which should include management of underlying comorbidities such as esophageal reflux or allergic rhinitis, dental evaluation for malocclusion, gingivitis, and dental caries, review of home medications (seizure medications), and less invasive therapies ranging from oral motor therapy and behavioral modifications to anticholinergic medications. In patients who fail more conservative management or with concerns for salivary aspiration, botulinum toxin injections and surgical procedures should be considered. Botulinum toxin injections function by chemical parasympathetic denervation, blocking the release of acetylcholine from pre-synaptic vesicles [2]. The effect of botulinum toxin on drooling was first demonstrated in patients with Parkinson’s disease in 1999; this principle has since been applied in the treatment of patients with sialorrhea secondary to neurologic disorders [3]. The objectives of surgical interventions include either reducing the flow of saliva through ligation of salivary ducts, or eliminating saliva through the removal of salivary glands.

Although many pediatric otolaryngologists treat sialorrhea within their patient populations, there exist few comprehensive reviews regarding practice patterns and outcomes. We, therefore, conducted a retrospective review of our clinical experience in treating sialorrhea at our institution over the last 10 years to determine which procedures were most commonly performed and their respective efficacy in decreasing sialorrhea. The review also examines varying treatment strategies and outcomes for patients with anterior and posterior drooling.

## Materials and methods

Study population

The institutional review board (IRB) approved a retrospective chart review of patients from Boston Children’s Hospital (BCH). The study population consisted of all patients less than 25 years of age who underwent a procedure for drooling (Current Procedural Terminology (CPT) codes 42440, 42450, 42509, 42510, 64611 matched with the International Classification of Diseases (ICD)-9 and ICD-10 codes 527.7 and K11.7) from January 1, 2006 to December 31, 2015. Ninety-three cases were identified involving 77 individual patients. Seventy-one patients were included in the final review after excluding individuals with a prior surgical intervention preceding the study date or patients lost to follow-up. The most commonly performed interventions for drooling included botulinum toxin injection into the parotid glands, submandibular glands (SMG), or both; bilateral sublingual gland excision with submandibular duct ligation +/- parotid duct ligation (SLGE and SMDL +/- PDL); submandibular gland excision (SMGE) +/- PDL; and SMDL or PDL alone or in combination. The procedures were performed by nine pediatric otolaryngologists within the group.

Data collection and measurements

We collected demographic information including patient age at intervention, gender, underlying diagnosis (i.e. cerebral palsy), drooling medications before and after surgery, and type of drooling (anterior, posterior, both). Clinical variables including type of procedure were also obtained from the medical record. Our outcome variables included pre- and post-procedure number of bibs, parent-reported outcomes, length of stay, and complications. Initially, data on episodes of pneumonia, antibiotic courses, and number of hospitalizations were collected, however, these data were not used as outcome measures due to patient complexity and frequency of antibiotic courses and hospital admissions secondary to co-morbidities not related to drooling. We defined improvement of symptoms by parent-reported outcomes, pre and post-procedure number of bibs, and need for a drooling medication for following the procedure. 

Statistical analysis

We compared the characteristics of patients who received botulinum injection vs. surgical intervention using Fisher’s exact test. Wilcoxon signed-rank test was used to compare the number of bibs before and after the procedure. All statistical analyses were performed using SAS version 9.4 (SAS Institute, Cary, NC, USA). The p-value significance used was 0.05.

## Results

Of the 71 patients included in the review, 43 patients (61%) were males, while 28 were female (39%). Forty (56%) were patients with cerebral palsy (Table 1). Three patients were over 18 years old.

**Table 1:** Pediatric patients with chronic sialorrhea SMG, submandibular gland; PG, parotid gland; SLGE, sublingual gland excision; SMDL, submandibular duct ligation; SMGE, submandibular gland excision.

	Botulinum toxin injection (n=28)	Surgical intervention (n=43)	
	n (%) or mean (SD)	n (%) or mean (SD)	p-value
Age, years, mean (SD)	7.9 (± 5.6)	9.6 (± 5.3)	0.12
Gender			0.08
Male	13 (46%)	30 (70%)	
Female	15 (54%)	13 (30%)	
Diagnosis			0.15
Cerebral palsy	19 (68%)	21 (49%)	
Neurologic disorders	9 (32%)	22 (51%)	
Medication (Robinul/Scopolamine)	19 (68%)	36 (84%)	0.15
Drooling			0.01
Anterior	21 (75%)	19 (44%)	
Posterior or both	7 (25%)	24 (56%)	
First procedure			
SMG botulinum toxin injection	22 (79%)	-	
SMG and PG botulinum toxin injection	6 (21%)	-	
SLGE with SMDL	-	25 (58%)	
SMGE	-	9 (21%)	
SMG and PG ligation	-	5 (12%)	
Others	-	4 (9%)	

The mean age at intervention was 8.9 (± 5.5) years, with a range of six months to 23 years. A total of 88 cases were evaluated; 28 patients who received botulinum toxin injection and 60 who received surgical intervention (43 patients as their first intervention, 14 patients following botulinum toxin injections, and three patients after tonsillectomy/adenoidectomy).

Thirty-one patients (44%) had posterior drooling or both anterior and posterior drooling. Those patients with posterior drooling were less likely to undergo botulinum toxin injections alone as the initial intervention (7/31; 23%) than patients with anterior drooling (21 botulinum toxin injections in 40 patients with anterior drooling; 53%; p=0.01). Approximately 58% of patients (18/31) with posterior drooling reported improvement, whereas 63% of patients (25/40) with anterior drooling reported improvement after the first intervention.

Of the 28 patients who received botulinum toxin injection as their first procedure, 22 had submandibular gland injections alone and six had both the submandibular and parotid glands injected. Twenty units of botulinum toxin was injected per gland in 22 cases (dosage range 5 units/gland-40 units/gland). All injections after 2012 were performed using ultrasound guidance (46%); bimanual palpation was used prior to 2012. Twenty-five patients had parent-reported outcome data available. Among this population, 56% (14/25) of caregivers and patients reported a subjective improvement; 74% (14/19) of patients on anticholinergic medications preoperatively continued the medication post-operatively. The reported number of bibs did not decline significantly (5.4 to 3.8 bibs, p = 0.4) (Table 2). Nine patients (32%) underwent botulinum toxin injections once, whereas 11 patients (39%) elected to continue with botulinum toxin injections alone as a second procedure. A total of 14 patients (50%) proceeded with surgical intervention (Table 2). 

**Table 2:** Patients who received botulinum toxin injection for their first intervention (n=28) SLE, sublingual excision; DL, duct ligation; SMG, submandibular gland; PG, parotid gland; SMGE, submandibular gland excision; SMD, submandibular duct.

Botulinum toxin injection for the first intervention	n (%)
1 botulinum toxin injection	9 (32%)
2 botulinum toxin injections	4 (14%)
1 botulinum toxin injection followed by surgical intervention	
SLE DL	3 (11%)
SMG and PG ligation	2 (7%)
SMGE	1 (4%)
SLE and SMG neurectomy	1 (4%)
1 botulinum toxin injection followed by 2 surgical interventions	
SLE DL then SMGE	1 (4%)
2 botulinum toxin injections followed by surgical intervention	
SLE DL	3 (11%)
SLE and SMD rerouting	1 (4%)
3 botulinum toxin injections followed by surgical intervention	
SMG and SMD ligation	1 (4%)
Tracheostomy	1 (4%)
4 botulinum toxin injections	1 (4%)

Among 60 patients who underwent surgical intervention, 36 patients had SLE with SMDL, 12 patients had SMGE, six patients underwent duct ligation (five patients 4 duct ligation; one patient SMDL), and six patients underwent other procedures (most commonly tonsillectomy/adenoidectomy). Forty-three operations were performed as the initial intervention whereas 17 of the procedures that were included in the analysis were performed following botulinum toxin injections or other procedures such as tonsillectomy +/- adenoidectomy. Two SMGE were performed following SLGE and these procedures were not included in the analysis given their history of prior salivary surgery. Overall, 73% of patients reported improvement after surgery. The mean number of bibs declined from 5.7 (SD 4.2) to 2.5 (SD 2.9) bibs after any surgical intervention (p<0.01). 

Caregivers noticed an improvement in drooling in 83% of patients following SMGE and 78% of patients after SLGE with SMDL (Table 3). 

**Table 3:** Botulinum toxin injection and surgical management of chronic sialorrhea

	Botulinum toxin injection (n=28)	SLGE with SMDL (n=36)	SMGE (n=12)	Ligation (n=6)	p-value
Age, years, mean (SD)	7.9 (± 5.6)	10.1 (± 4.8)	7.2 (± 5.5)	6.2 (± 4.4)	0.06
Medications prior to surgery	19 (68%)	29 (81%)	8 (67%)	5 (83%)	0.62
Anterior/posterior					0.01
Anterior	21 (75%)	23 (64%)	4 (33%)	2 (33%)	
Posterior or both	7 (25%)	13 (36%)	8 (57%)	4 (67%)	
Post-operative complication					
Overall (n=patients)	4 (15%)	9 (25%)	1 (8%)	3 (50%)	0.19
Sialadenitis	-	2 (6%)	-	1 (17%)	
Respiratory infection	-	1 (3%)	1 (8%)	-	
Dysphagia	1 (4%)	-	-	-	
Dehydration	1 (4%)	2 (6%)	-	-	
Thickened saliva	1 (4%)	-	-	-	
Others	1 (4%)	5 (14%)	-	2 (33%)	
Reported improvement	14/25 (56%)	28/36 (78%)	10/12 (83%)	3/6 (50%)	0.13
Reported number of bibs					
Pre-op	5.4 (± 4.1)	4.8 (± 2.8)	10.0 (± 10.6)	7.3 (± 3.9)	
Post-op	3.8 (± 3.3)	2.4 (± 3.6)	1.5 (± 0.0)	2.3 (± 0.4)	
p-value	p = 0.40	p < 0.01	-	-	
Continued medication after procedure	14/19 (74%)	16/29 (55%)	5/8 (63%)	3/5 (60%)	0.47
Required further intervention	19/28 (68%)	9/36 (25%)	2/12 (17%)	1/6 (17%)	<0.001

There was a statistically significant decrease in the number of bibs after SLGE and SMDL (4.8 to 2.4, p<0.01). While the decrease in the number of bibs following SMGE was 10 to 1.5, there was not enough data to measure significance. Of those patients who were on medication pre-operatively, 57% (24/42 patients) required anticholinergic medication post-operatively.

After any intervention, complications were reported in 17 patients (28%) (Table 2). SLGE with SMDL accounted for the majority of complications (9/17 patients, 10/18 reported complications). The most commonly reported complications were sialadenitis (5%), respiratory infections (3%), dehydration (5%), and 13% experienced other complications (increased seizures, Clostridium difficile infection, oral thrush). The risk of complications varied by intervention, though we did not find a statistically significant difference (p=0.19) in complication rates between interventions.

## Discussion

This investigation represents a large sample size of pediatric patients with neurologic impairment and chronic sialorrhea treated with botulinum toxin, surgical management, or both. We retrospectively reviewed our institutional experience with sialorrhea and found that patients with posterior drooling were significantly more likely to undergo surgical interventions following failure of conservative measures and anticholinergic medications. Not surprisingly, surgical interventions with the exception of 2 or 4 duct ligation also provided a more definitive treatment option.

Prior studies have assessed the use of botulinum toxin injections or various surgical treatments separately [3-7]. Formeister et al. published a review of 27 pediatric patients which included both botulinum toxin injections and surgery as treatment options [8]. They also found improved outcomes following surgical interventions versus botulinum toxin injections. The authors concluded that surgical interventions were overall more effective in controlling drooling than botulinum toxin injections and advocated for earlier surgical intervention. In our patient population, we found that otolaryngologists were more likely to perform botulinum toxin injections as the initial intervention following failed conservative or medical management in patients with anterior drooling than in those with posterior drooling. This is likely related to the severe consequences of ongoing posterior drooling.

Patient and/or caregiver-reported improvement following botulinum toxin injections was 56%, although the reported number of bibs did not decline significantly. There were four reported complications, including self-resolving dysphagia, thickening of secretions, and re-admission for dehydration. This 15% complication rate is comparable to the wide range published in the literation, 0.6%-24%. The degree of improvement noted within our population is also similar to the 42%-68% range that has been cited [5,6,9]. Forty-three percent of the patients in our sample underwent additional botulinum toxin injections, which is equivalent to Formeister’s study [8]. Fifty-four percent of the patients who received botulinum toxin in this study were eventually treated surgically, with the majority reporting improvement after surgery. 

Surgical interventions, although more invasive, not surprisingly provide a more definitive treatment option for sialorrhea. SMGE and SLGE with SMDL each demonstrated reported improvements greater than botulinum toxin injections, although 4 duct ligation was less effective among our population (50% vs. 56%). We observed a significant decline in the mean number of bibs following surgical intervention.

Patients with posterior drooling were more likely to be managed with surgical interventions. SLGE with SMDL was the most commonly performed procedure. This procedure has not been described in the literature, but from a procedural perspective is similar to submandibular duct rerouting (SMDR) with SLGE. The advantage in our population, however, is that it is not limited to patients with an intact swallow, but can be safely performed in children who aspirate since the ducts are ligated and not rerouted. Greensmith et al. reviewed outcomes of 72 patients who underwent SMDR/SLE, 58.2% of patients had an improvement in drooling frequency and 77.6% had an improvement in their drooling-severity score [10]. Similarly, 72% of our patients and caregivers reported improvement following SLGE with SMDL. SMGE was similarly associated with an improvement in drooling in 78% of patients. This is consistent with what has been reported, with Osorio et al. reporting infrequent or absence of drooling postoperatively in 55.3% of cases and occasional drooling in 31.9% of cases; Delsing et al. published a 63% overall response rate (50% reduction in drooling quotient) with SMGE alone [11,12]. Dundas and Peterson had greater success with the addition of PDL, with a good to excellent response rate in more than 85% of patients [13]. The number of patients undergoing duct ligation in our review was small (six patients) making it difficult to derive conclusions from the data. While this review does not allow us to make conclusions on which surgical intervention is preferred, it does confirm that surgical management continues to have good success rates and can provide solutions for patients with sialorrhea refractory to medication, botulinum toxin injections, and in cases of posterior drooling.

There are several limitations to this retrospective review, including the small patient numbers within the various treatment arms. The study also carries the limitations inherent to retrospective reviews with the results being dependent on physician charting and parental reporting. By not using an objective measure, such as a standard grading scale, the results are dependent on parental impression and may be influenced by parental bias. This does, however, keep with the larger trend of patient-reported outcomes, with some arguing that these outcomes are more relevant and are precisely the ones that should be collected [14,15]. The last limitation is that the data points were collected at varying times during the recovery. There was no consistency in the timing and length of patient follow-up. Despite the variability in the data collection, the results within this study are similar to those previously reported in the literature, which reinforces validity to the findings. Future reviews will incorporate the use of the teacher drooling scale, a validated scale used to measure anterior drooling [16].

While several retrospective reviews examining the management of drooling have been published, this review examines the variability in the approach for patients with anterior or posterior drooling and examines how outcomes may vary between these populations. Additionally, we examined the roles of botulinum toxin injections and surgical interventions in drooling management, as long-term care often involves an interplay between both interventions.

## Conclusions

This retrospective review on the management of sialorrhea with botulinum toxin injections and surgical interventions demonstrated several key findings, including that patients with posterior drooling have an increased likelihood of initial surgical intervention. Due to the small sample size we were unable to conclude if there is a statistically significant difference in complication rates between interventions. While management of this population continues to be difficult as no intervention provides certainty for improvement, these findings emphasize the importance of coordinated care in this population to help manage caregiver expectations and to discuss the potential need for additional interventions or continued use of medications following any intervention.
